# Finding value and beauty in obsolete scientific equipment

**DOI:** 10.1038/s44319-024-00057-1

**Published:** 2024-01-17

**Authors:** David R Smith

**Affiliations:** Western University, London, ON Canada

**Keywords:** History & Philosophy of Science, Methods & Resources

## Abstract

Most scientific equipment gets heedlessly thrown away when it’s no longer of use. But these often beautiful and well-crafted tools deserve a better fate, if anything to remind younger generations of the technological and scientific breakthroughs that they enabled.

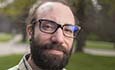

Every passion borders on the chaotic, but the collector’s passion borders on the chaos of memories.

Walter Benjamin

Growing up in northern Ontario, I realized early on that my mom was an antique-aholic. She would drag me kicking and screaming to every garage sale, flea market, and thrift store within a 200 km radius. On family road trips if I covertly spotted a sign for antiques, I’d point the other way and yell “bear”, hoping to distract my mother. But she had a sixth sense for dilapidated things and even a celebration of polar bears would not have prevented us from driving down some washed-out dirt road and spending three hours discussing the finer points of Wedgwood pottery inside an eighteenth-century barn. “Please,” I’d beg, “can we just go home.” After countless whacks across the back of the head, I came to realize that my only hopes were vintage Playboy, O-Pee-Chee hockey cards and the illustrious cowboy holster.

I have learnt the hard way that one person’s trash is another person’s treasure. Fortunately for me, university biology departments are an excellent place to put this idea into practice. Over the years, I’ve rescued many timeworn scientific instruments from garbage bins. Some of these items include tarnished balanced scales, 100-year-old brass microscopes, creaky oak cabinets, miniature glassware, laboratory mechanical stopwatches, 35-mm microscope film cameras, eclectic cardboard file holders, unusual technical manuals, and an endless array of wooden slide holders. Are they worth anything? Let’s just say the whole kit and kaboodle would not get me a pair of original Staffordshire spaniel dog figurines in pristine condition. Or, as my mom would politely put it: “Dear boy, have I not taught you anything? Never choose quantity over quality.”

Call me a scientific hoarder, but I do find deep meaning, inherent value, and beauty in these scientific artifacts. Take, for instance, my most recent trash-heap find: a fifty-five-year-old Kensco Artificial Population Sampler. This massive, table-sized instrument was designed for teaching the principles of sampling and the measurement of populations. The large plexiglass sheets, which form the surface of the table, are like works of contemporary art, containing hundreds of circles of varying size and color. And the accompanying sampling tools look like something a Swiss watchmaker would have at their workbench. Like any good collector, I have the original box and papers, including a hand-typed and signed letter from the actual inventor of the device, Arnold M. Schultz. It reads: “By now, I presume you have tested your Population Sampler in the classroom and laboratory. I would indeed appreciate some frank comments on how you feel about the apparatus, student response to learning, and any criticism you would like to make.” Can you imagine receiving a letter like this from the CEO of Qiagen with your next DNA extraction kit? Looking at it today, you might think that this now-obsolete population sampler was an inexpensive purchase. But in 1967, it cost two grand, which is equivalent to $18,000 today, when factoring in inflation. Still, I don’t think I could give away this cumbersome device, so it sits at the back of my office until I too make my way to the proverbial university trash heap.

It seems sad, wasteful, and shortsighted to dispose of equipment that was once worth tens of thousands of dollars. But the world of science is driven by novelty—as it should be—and as any academic can attest, space on university campuses is at a premium. Indeed, we don’t need fridge-sized Sanger sequencers cluttering up our labs when pocket-sized MinIONs are available. What will become of all these obsolete sequencing machines, many of which cost more than a Ferrari?

I think it would be cool to have them on display in the lobbies of science departments, for example, with brief descriptions about their history and the various landmark genomes they helped to sequence. Moreover, many of the early Applied Biosystem sequencers have a gorgeous neo-vintage sci-fi aesthetic, like something out of the Alien movie franchise—but I think university administrations are going for a different kind of look. Do not forget, as well, that old pieces of technology that seem worthless today sometimes becomes valuable a short while later. Two years ago, for instance, a pristine and unopened copy of the 1996 video game *Super Mario 64* sold for $1.56 million at auction. I imagine that early production copies of the first automated DNA sequencer (the AB370A) will soon become collectors’ items, if they have not already.

Valuable or not, nothing livens up a sterile and clinical university office like some good, old-fashioned scientific memorabilia. My office, you won’t be surprised to read, is thoughtfully decorated—that is, disorderly cluttered—with antiquated research bric-a-brac, such as old wooden microscope boxes, retired pipettors, brass instruments of varying size and function, and faded botanical prints, giving it a Hogwarts kind of vibe. When students stop by for office hours, they get a kick out of all the bizarre stuff, and I think it adds to their university experience. My wife, on the other hand, doesn’t appreciate that my office aesthetic has diffused to our home environment as well. Every year or two, I take a few snapshots of my office décor and send them to my mother who always hones in on the most interesting piece and asks if she can trade me something for it.

Although she’ll soon be eighty, my mom still runs a small antique shop in Mahone Bay, Nova Scotia. Whenever I visit, I walk around the store taking in the history and grandeur of the items, from kitschy costume jewelry to art deco scent bottles to Biltmore felt hats. The life scientist in me always reaches for the taxidermy-type stuff, like scrimshaw, Victorian scarab beetle brooches and *Hirschgrandln*, which is a type of 19th-century German jewelry set with stag’s teeth, a gift from the hunter to his sweetheart. Thinking of my mom reminds me that there is an art to collecting and curating. As scientists, we understand the drive to collect and categorize biological specimens, and we value the beauty of the natural world. But sometimes, we fall short of appreciating the inherent value and splendor in inanimate objects, be it an old dissection kit or a sequencing machine.

So, the next time you walk into a lab, take a deep breath, and have a good look around at all that hardworking, magnificent equipment, big and small, old and new. Listen closely to the hum of the minus 80 freezer, the 27-inch monitors, the floor centrifuge, the fume hood, and the growth chamber. Can you hear it? The whisper of hundreds of research tools chanting in unison: “Please do not send me to the junkyard. Please do not Marie Kondo me. I promise that when I’m no longer churning out data and contributing to high-impact papers, I’ll sit quietly in your office, garage, or basement preserving the memories of research past.” If my mom is not proof enough, then take it from poet, playwright, and novelist Johann Wolfgang von Goethe who said it best: “Collectors are happy people.”

